# Buffalo Pathological Society

**Published:** 1889-11

**Authors:** Eugene A. Smith

**Affiliations:** Secretary


### BUFFALO PATHOLOGICAL SOCIETY.

### REGULAR MEETING, SEPTEMBER 20, 1889.

Reported by EUGENE A. SMITH, M. D., Secretary.

Dr. JAMES W. PUTNAM read a paper entitled "The Graver Forms of Hysteria." (See October number, page 153.)

Dr. JEWETT asked the essayist for the differentiating points between hysteria and chorea.

Dr. PUTNAM mentioned the history of the case, the sudden onset in hysteria, the presence of one or more spots of anesthesia, and the fact that hysteric patients can be hypnotized.

Dr. JEWETT also asked if genital troubles had a causative effect on hysteria.

The answer was a qualified No. The essayist said, "We press over the ovaries, in hysteria, because they are sensitive points."

Dr. STOCKTON related the history of a rheumatic family. The three children, successively, had chorea, and one had abnormal heart sounds.

Dr. PRYOR wished to find out if hysterical cases ever had the endocarditis, with peculiar bead-like vegetations on the heart valves, commonly seen in chorea.

Dr. PARK told of a young woman who developed spinal trouble after mental disturbance. The case had passed through several physicians' hands undiagnosticated. Dr. Park and Dr. Putnam had found her with a hyperesthetic spine, inframammary point, and left ovary. The case was diagnosticated and successfully treated as hysterical.

Dr. PARMENTER told of a patient, 60 years old, who fell when a young woman and dislocated her ankle. Since then, the foot has been extremely sensitive, and the sensitiveness has spread gradually up to the groin. There is no organic change discoverable.

Dr. PRYOR desired to be told the difference between hysterical coma and trance. He saw a man at the Fitch Hospital who had general anesthesia, staring, widely-opened eyes; breathing and pulse, normal. Upon having ice-water dashed in his face, and a repetition of the dose threatened, he rose and walked off. A similar case at the General Hospital walked away rather than take a dose of castor oil, which was ordered in her hearing.

Dr. PUTNAM said that pressure over the ovaries will always wake a woman from hysterical trance. Replying to another question, Dr. Putnam said that most trance-sleepers are fairly well nourished, but he had seen one who starved herself.

Dr. VAN PEYMA asked for a definition of hysteria, and Dr. Putnam stated that he could not give a clear definition. He said that specialists differ, and that the best he can do is to call it a nervous disease which cannot be accounted for by pathological changes, and which responds to certain symptoms.

Dr. STOCKTON, under Voluntary Communications, gave the following report of a case at the General Hospital: A mulatto was brought in, dull, muttering in delirium, and without obtainable history. His eyes were slightly sensitive, pupils contracted; it was difficult for him to swallow, and his abdomen was contracted. He had typhoidal diarrhea. His temperature ranged between 101° and 103°. The *post mortem* revealed ℥iv. of bloody serum in his left pleura, the endocardium had a peculiar bluish appearance, and one cusp of the mitral valve was perforated by an ulcer the size of a split pea. In the opening of the ulcer was a blood-clot. The small intestine, four feet above the ileo-cecal valve, was inflamed and contained ochre-colored feces. The spleen was soft and pulpy in its upper two-thirds. The kidneys contained several old caseated infarcts and a few fresh ones. In the brain were two fresh spots of infarction.

Dr. PRYOR asked that microscopical sections of the ulcer be made, to find if it is a case of septic endocarditis, as described by Prudden.

Dr. STOCKTON reported a case of emphysema extending into the subcutaneous cellular tissue over the whole trunk. The child died of acute miliary tuberculosis, and a *post mortem* revealed a perforating ulcer of the right bronchus, permitting air to work along the trachea and over the trunk.

Dr. PARK described a case in which the air escaped at the spot of a broken rib, and extended over the trunk and down on the thigh. He compared it to a butcher blowing up the subcutaneous cellular tissue to make slaughtered animals appear fat.

Dr. PARMENTER reported a case similar to Dr. Stockton's. Dr. Stockton stated that "Pip" in chickens is emphysema, caused by a worm which perforates the trachea.

Dr. VAN PEYMA told of a case of labor, in which, head and shoulders being born, the child was delivered with difficulty. Owing to abdominal dropsy, the abdomen measured eighteen inches in circumference.

Dr. PRYOR reported a case of syphilitic pneumonia which cleared up after treating the patient with iodide of potassium. He diagnosticated the trouble because the part affected was the middle lobe of the left lung, and the patient had ulceration of the tonsils. A feature in this case, not usually seen in such cases, was the presence of fever, the temperature at times reaching 103°.

Dr. BERGTOLD reported that to kill a great horned owl, weight four pounds, he injected one and a half grains of morphine into its peritoneum without seeming to dull or even affect the bird.

In answer to the questions propounded at the August meeting, Dr. PARMENTER declared himself unable to give the etiological effect of alcoholism in the parents on congenital umbilical hernia, and in regard to mortality, he said that congenital cases are commonly fatal, only fifty per cent. of the less severe cases recovering under the use of antiseptic compresses.

Dr. PARK reported a case of cholecystotomy, performed on a jaundiced woman who had an abdominal tumor containing pus and a history of passage of biliary calculi. One hundred and four gallstones were removed and the sac drained. The patient is doing well.

Dr. JEWETT contrasted this case with one he had seen twelve years ago, when, with the best medical men in the country in attendance, the patient died without an attempt at operative relief.

### STATED MEETING, OCTOBER 18, 1889.

Dr. PARK presented a specimen of recent fracture of the shaft of the humerus, removed from the body of an aged man, whose death followed some twelve days after the injury. It exhibited recent callus, small in amount, but normal in arrangement, and was shown simply as an example of a normal condition seldom seen in recent specimens.

The same case exhibited another condition of extreme interest. The patient developed before his death a traumatic mania to such an extent as to require restraint. During this he displayed a spastic paralysis of the right leg. There was loss of motion and sensation of the foot, yet the muscles were in a condition of spastic spasm. There was a difficulty of diagnosis, as between injury to the limb and some recent or old cerebral lesion, especially in the absence of any history. It was held that the latter was the probable condition.

Upon autopsy a localized area of congestion was found directly over the cortical center of the right leg. Around and beneath this was a wider area of edema, both of the pia and the cortex. Careful examination of the underlying parts of the brain failed to reveal any explanation of the lesion, either in the presence of emboli or any other disturbances.

